# Supplementary material for: Data-driven prediction of future purchase behavior in cross-border e-commerce using sequence modeling with PSO-tuned LSTM
Source: PLoS One. 2025 Dec 10;20(12):e0337932. doi: 10.1371/journal.pone.0337932 (PMC12799269; doi:10.1371/journal.pone.0337932)
Supplement: S1 — (PDF) [file pone.0337932.s001.pdf]

```
tic

clc

clear all

fs=1; %Sampling frequency, which refers to the time interval between two data points in a time series, with an interval of 1 hour for sampling

Ts=1/fs; %Sampling period


X=xlsvead ('voltage. xlsx ','A1: A720');


save origin_data X


L=length(X); %Number of sampling points, i.e. how many data points are there

t=(0:L-1)*Ts; %Time series

STA=0; %Sampling starting position, where sampling starts at the 0th hour


%---------Some sample parameters for VMD: Setting VMD sample parameters---------------

alpha = 2500; %Moderate bandwidth constraint: a moderate bandwidth constraint/penalty factor

tau = 0; %Noise tolerance (no strict fidelity enforcement): noise tolerance (no strict fidelity enforcement)

K = 5; %Modes: the number of decomposed modes

DC = 0; %No DC part exposed: No DC part

init = 1; %Initialize omegas uniformly: uniform initialization of omegas

tol = 1e-7

%---------------Run actual VMD code: Perform VMD decomposition on data---------------------------

[u, u_hat, omega] = VMD(X(:,end), alpha, tau, K, DC, init, tol);
```

```
save vmd_data u

figure(1);
imfn=u;
n=size(imfn,1);    %size(X,1), Return the number of rows in matrix X; size(X,2), Return the number of columns in matrix X; N=size(X,2),     It is to assign the number of columns in matrix X to N
subplot(n+1,1,1); %M represents rows, n represents columns, and p represents the row and column on which graph to draw. For example, subplot (2,2, [1,2])
plot(t,X(:,end));
ylabel('Original','fontsize',10);
% title('VMD Decomposition');

for n1=1:n
subplot(n+1,1,n1+1);
plot(t,u(n1,:)); %Output IMF components, where a (:, n) represents the nth column element of matrix a, and u (n1,:) represents the n1 row element of matrix u
ylabel(['IMF' int2str(n1)]); %Int2str (i) is the value of i rounded to the nearest integer and converted to a character, named on the y-axis
end
xlabel('Time(s)','fontsize',12);


%Sparrow Optimization Algorithm%
%_____________________________________________________________________%
function [Best_pos,Best_score,curve,BestNet]=SSA(pop,Max_iter,lb,ub,dim,fobj)
Disp (sparrow algorithm starts) ..')
```

```
ST = 0.6; %Warning value
PD = 0.7; %The ratio of discoverers, the rest are joiners
SD = 0.2; %Realizing the proportion of dangerous sparrows

PDNumber = round(pop*PD); %Number of discoverers
SDNumber = round(pop*SD); %Realizing the dangerous sparrow population
if(max(size(ub)) == 1)
ub = ub.*ones(1,dim);
lb = lb.*ones(1,dim);
end
net = {};
%Population initialization
X0=initialization(pop,dim,ub,lb);
X = X0;
%Calculate the initial fitness value
fitness = zeros(1,pop);
for i = 1:pop
[fitness(i),net{i}] =   fobj(X(i,:));
end
[fitness, index]= sort(fitness); %Sort by
BestF = fitness(1);
WorstF = fitness(end);
GBestF = fitness(1); %Global optimal fitness value
for i = 1:pop
X(i,:) = X0(index(i),:);
net{i}=net{index(i)};
end
```

```
curve=zeros(1,Max_iter);

GBestX = X(1,:); %Global optimal position

X_new = X;

BestNet = net{1};

curve(1)=GBestF;

for i = 2: Max_iter

Disp (['di ', num2str(i), 'iteration']);

BestF = fitness(1);

WorstF = fitness(end);



R2 = rand(1);

for j = 1:PDNumber

if(R2<ST)

X_new(j,:) = X(j,:).*exp(-j/(rand(1)*Max_iter));

else

X_new(j,:) = X(j,:) + randn()*ones(1,dim);

end

end

for j = PDNumber+1:pop

%           if(j>(pop/2))

if(j>(pop - PDNumber)/2 + PDNumber)

X_new(j,:)= randn().*exp((X(end,:) - X(j,:))/j^2);

else

%Generate a random number of -1,1

A = ones(1,dim);

for a = 1:dim
```

```
if(rand()>0.5)

A(a) = -1;

end

end

AA = A'*inv(A*A');

X_new(j,:)= X(1,:) + abs(X(j,:) - X(1,:)).*AA';

end

end

Temp = randperm(pop);

SDchooseIndex = Temp(1:SDNumber);

for j = 1:SDNumber

if(fitness(SDchooseIndex(j))>BestF)

X_new(SDchooseIndex(j),:) = X(1,:) + randn().*abs(X(SDchooseIndex(j),:) - X(1,:));

elseif(fitness(SDchooseIndex(j))== BestF)

K = 2*rand() -1;

X_new(SDchooseIndex(j),:) = X(SDchooseIndex(j),:) + K.*(abs( X(SDchooseIndex(j),:) -
X(end,:))./(fitness(SDchooseIndex(j)) - fitness(end) + 10^-8));

end

end

%Boundary control

for j = 1:pop

for a = 1: dim

If (X_new (j, a)>ub (a) | | isnan (X_new (j, a))% sNaN() function is used to determine
whether a value is NaN. If it is NaN, the upper boundary value is taken

X_new(j,a) =ub(a);

end

if(X_new(j,a)<lb(a)||isnan(X_new(j,a)))
```

```
X_new(j,a) =lb(a);

end

end

end

%Update location

for j=1:pop

[fitness_new(j),net{j}] = fobj(X_new(j,:));

end

for j = 1:pop

if(fitness_new(j) < GBestF)

GBestF = fitness_new(j);

GBestX = X_new(j,:);

BestNet=net{j};

end

end

X = X_new;

fitness = fitness_new;

%Sort Update

[fitness, index]= sort(fitness); %Sort by

BestF = fitness(1);

WorstF = fitness(end);

for j = 1:pop

X(j,:) = X(index(j),:);

net{j}=net{index(j)};

end

curve(i) = GBestF;

end
```

```
Best_pos =GBestX;

Best_score = curve(end);

end


function [gbest,g,Convergence_curve]=PSO(N,T,lb,ub,dim,fobj)

%%Define the parameters of particle swarm optimization algorithm

%N population T iteration times

%%Randomly initialize the population

D=dim; %Particle dimension

c1=1.5; %Learning Factor 1

c2=1.5; %Learning Factor 2

w=0.8; %Inertial weight


Xmax=ub; %Maximum position value

Xmin=lb; %Minimum position value

Vmax=ub; %Maximum speed

Vmin=lb; %Minimum speed value

%%

%%%%%%%%%%%%%%Initialize population individuals (limited position and velocity)%%%%%%%%%%%%


x=rand(N,D).*(Xmax-Xmin)+Xmin;

v=rand(N,D).*(Vmax-Vmin)+Vmin;

%%%%%%%%%%%%%%%%Initialize individual optimal position and optimal value%%%%%%%%%%%%%%%

p=x;

pbest=ones(N,1);
```

```
for i=1:N

pbest(i)=fobj(x(i,:));

end

%%%%%%%%%%%%%%%%%%%Initialize the global optimal position and optimal value%%%%%%%%%%%%%%%

g=ones(1,D);

gbest=inf;

for i=1:N

if(pbest(i)<gbest)

g=p(i,:);

gbest=pbest(i);

end

end

%%%%%%%%%%%Iterate sequentially according to the formula until the accuracy or number of iterations%%%%%%%%%%

for i=1:T

i

for j=1:N

%%%%%%%%%%%%%%Update individual optimal position and optimal value%%%%%%%%%%%%%%%

if (fobj(x(j,:))) <pbest(j)

p(j,:)=x(j,:);

pbest(j)=fobj(x(j,:));

end

%%%%%%%%%%%%%%%%Update the global optimal position and optimal value%%%%%%%%%%%%%%

if(pbest(j)<gbest)

g=p(j,:);

gbest=pbest(j);
```

```
end
%%%%%%%%%%%%%%%Follow the new position and speed values%%%%%%%%%%%%%%%%%%%
v(j,:)=w*v(j,:)+c1*rand*(p(j,:)-x(j,:))...
+c2*rand*(g-x(j,:));
x(j,:)=x(j,:)+v(j,:);
%%%%%%%%%%%%%%%%%%%Boundary condition processing%%%%%%%%%%%%%%%%%%%%%
if length(Vmax)==1
for ii=1:D
if (v(j,ii)>Vmax)  |  (v(j,ii)< Vmin)
v(j,ii)=rand * (Vmax-Vmin)+Vmin;
end
if (x(j,ii)>Xmax)  |  (x(j,ii)< Xmin)
x(j,ii)=rand * (Xmax-Xmin)+Xmin;
end
end
else
for ii=1:D
if (v(j,ii)>Vmax(ii))  |  (v(j,ii)< Vmin(ii))
v(j,ii)=rand * (Vmax(ii)-Vmin(ii))+Vmin(ii);
end
if (x(j,ii)>Xmax(ii))  |  (x(j,ii)< Xmin(ii))
x(j,ii)=rand * (Xmax(ii)-Xmin(ii))+Xmin(ii);
end
end
end


end
```

```
%%%%%%%%%%%%%%%%%%%Record the global optimal value of each generation%%%%%%%%%%%%%%%%%%%%

Convergence_curve(i)=gbest; %Record the fitness value of the training set

disp(['current iteration is: ',num2str(i), ', best fitness is: ', num2str(gbest)]);


end

clc;

clear

close all


%%LSTM prediction

tic

load origin_data.mat

load vmd_data.mat


disp('…………………………………………………………………………………………………………………')

Disp ('Single LSTM Prediction')

disp('…………………………………………………………………………………………………………………')


num_samples = length(X); %Number of samples

kim = 5; %Delay step size (Kim historical data as independent variable)

zim =   1; %Predicting across Zim time points

or_dim = size(X,2);


%Refactoring the dataset

for i = 1: num_samples - kim - zim + 1

res(i, :) = [reshape(X(i: i + kim - 1,:), 1, kim*or_dim), X(i + kim + zim - 1,:)];
```

```
end



%Division of training and testing sets
outdim = 1; %The last column is the output
num_size = 0.7; %Proportion of training set to dataset
num_train_s = round(num_size * num_samples); %Number of training set samples
f_ = size(res, 2) - outdim; %Input feature dimension



P_train = res(1: num_train_s, 1: f_)';
T_train = res(1: num_train_s, f_ + 1: end)';
M = size(P_train, 2);

P_test = res(num_train_s + 1: end, 1: f_)';
T_test = res(num_train_s + 1: end, f_ + 1: end)';
N = size(P_test, 2);

%Data normalization
[p_train, ps_input] = mapminmax(P_train, 0, 1);
p_test = mapminmax('apply', P_test, ps_input);

[t_train, ps_output] = mapminmax(T_train, 0, 1);
t_test = mapminmax('apply', T_test, ps_output);

%Format conversion
for i = 1 : M
```

vp_train{i, 1} = p_train(:, i);

%The reconstructed data has 24 inputs and 1 output, totaling 1571 lines. Through this format conversion,

%When i=1, pack the 24 inputs from the first row into a 1 * 1 cell package,

%And so on, it becomes a total of 1571 cells, because {i, 1}, these 1571 cells are arranged in a column

%And the 24 numbers inside one cell are arranged vertically by 24 * 1

%The data format of the cell is double

vt_train{i, 1} = t_train(:, i);

end

for i = 1 : N

vp_test{i, 1} = p_test(:, i);

vt_test{i, 1} = t_test(:, i);

end

%Create an LSTM network,

layers = [ ...

SequenceInputLayer (f_)% Input Layer

lstmLayer(70)

reluLayer

Fully ConnectedLayer (outdim)% Regression Layer

regressionLayer];

%Parameter settings

Options=trainingOptions ('adam ',...% optimization algorithm Adam

'MaxEpochs', 70, . ..% maximum training times

```
'GradientThreshold',    1, . ..% gradient threshold
'InitialLearnRate',    0.01, . ..% initial learning rate
'LearnRateSchedule', 'piecewise',    . Adjustment of learning rate
'LearnRateDropPeriod',    60, . Adjust learning rate after 850 training sessions
'LearnRateDropFactor', 0.2, . ..% learning rate adjustment factor
'L2Regularization',    0.01, . ..% regularization parameter
'ExecutionEnvironment', 'cpu', ...% Training Environment
'Verbose', 0 ..% Close optimization process
'Plots', 'training-progress'); %Draw a curve

%Training
net = trainNetwork(vp_train, vt_train, layers, options);
%analyzeNetwork(net); %View network structure
%Prediction
t_sim1 = predict(net, vp_train);
t_sim2 = predict(net, vp_test);

%Data de normalization
T_sim1 = mapminmax('reverse', t_sim1, ps_output);
T_sim2 = mapminmax('reverse', t_sim2, ps_output);
T_train1 = T_train;
T_test2 = T_test;

%Data format conversion
T_sim1 = cell2mat(T_sim1); %Cell2mat converts cell arrays into regular arrays
T_sim2 = cell2mat(T_sim2);
```

```
%Indicator calculation
Disp ('Training Set Error Indicator')
[mae1,rmse1,mape1,error1]=calc_error(T_train1,T_sim1');
fprintf('\n')

Disp ('Test Set Error Indicator')
[mae2,rmse2,mape2,error2]=calc_error(T_test2,T_sim2');
fprintf('\n')
toc



tic
disp('…………………………………………………………………………………………………………………………')
Disp (VMD-LSTM prediction)
disp('…………………………………………………………………………………………………………………………')

imf=u;
c=size(imf,1);
%%Modeling each component
for d=1:c
Disp (['di ', num2str(d), 'Component Modeling'

X_imf=[X(:,1:end-1) imf(d,:)'];
num_samples = length(X_imf); %Number of samples

%Refactoring the dataset
for i = 1: num_samples - kim - zim + 1
```

```
res(i, :) = [reshape(X_imf(i: i + kim - 1,:), 1, kim*or_dim), X_imf(i + kim + zim - 1,:)];
end



%Division of training and testing sets
outdim = 1; %The last column is the output
num_size = 0.7; %Proportion of training set to dataset
num_train_s = round(num_size * num_samples); %Number of training set samples
f_ = size(res, 2) - outdim; %Input feature dimension



P_train = res(1: num_train_s, 1: f_)';
T_train = res(1: num_train_s, f_ + 1: end)';



P_test = res(num_train_s + 1: end, 1: f_)';
T_test = res(num_train_s + 1: end, f_ + 1: end)';



%Data normalization
[p_train, ps_input] = mapminmax(P_train, 0, 1);
p_test = mapminmax('apply', P_test, ps_input);

[t_train, ps_output] = mapminmax(T_train, 0, 1);
t_test = mapminmax('apply', T_test, ps_output);

%Format conversion
```

```
for i = 1 : M

vp_train{i, 1} = p_train(:, i);

vt_train{i, 1} = t_train(:, i);

end


for i = 1 : N

vp_test{i, 1} = p_test(:, i);

vt_test{i, 1} = t_test(:, i);

end


%Create an LSTM network,

layers = [ ...

SequenceInputLayer (f_)% Input Layer

LstmLayer (70)% LSTM layer

ReluLayer% Relu activation layer

Fully ConnectedLayer (outdim)% Regression Layer

regressionLayer];


%Parameter settings

Options=trainingOptions ('adam ',...% optimization algorithm Adam

'MaxEpochs',   70, . ..% maximum training times

'GradientThreshold',   1, . ..% gradient threshold

'InitialLearnRate',   0.01, . ..% initial learning rate

'LearnRateSchedule', 'piecewise',   . Adjustment of learning rate

'LearnRateDropPeriod',   60, . Adjust learning rate after 850 training sessions

'LearnRateDropFactor', 0.2, . ..% learning rate adjustment factor

'L2Regularization',   0.01, . ..% regularization parameter
```

```
'ExecutionEnvironment', 'cpu', ...% Training Environment
'Verbose', 0 ..% Close optimization process
'Plots', 'training-progress'); %Draw a curve

%Training
net = trainNetwork(vp_train, vt_train, layers, options);
%Prediction
t_sim5 = predict(net, vp_train);
t_sim6 = predict(net, vp_test);

%Data de normalization
T_sim5_imf = mapminmax('reverse', t_sim5, ps_output);
T_sim6_imf = mapminmax('reverse', t_sim6, ps_output);

%Data format conversion
T_sim5(d,:) = cell2mat(T_sim5_imf); %Cell2mat converts cell arrays into regular arrays
T_sim6(d,:) = cell2mat(T_sim6_imf);
T_train5(d,:)= T_train;
T_test6(d,:)= T_test;
end

%Add up the predicted results of each component
T_sim5=sum(T_sim5);
T_sim6=sum(T_sim6);
T_train5=sum(T_train5);
T_test6=sum(T_test6);
```

```
%Indicator calculation
Disp ('Training Set Error Indicator')
[mae5,rmse5,mape5,error5]=calc_error(T_train5,T_sim5);
fprintf('\n')

Disp ('Test Set Error Indicator')
[mae6,rmse6,mape6,error6]=calc_error(T_test6,T_sim6);
fprintf('\n')
toc

%%VMD-SSA-LSTM prediction
tic
disp('……………………………………………………………………………………………………………')
Disp (VMD-SSA-LSTM prediction)
disp('……………………………………………………………………………………………………………')

%SSA parameter settings
pop=3; %Population size
Max_iter=5; %Maximum number of iterations
dim=3; %Optimize the three parameters of LSTM
lb = [50,50,0.001]; %Lower boundary
ub = [300,300,0.01]; %Upper boundary
numFeatures=f_;
numResponses=outdim;
fobj = @(x) fun(x,numFeatures,numResponses,X) ;
[Best_pos,Best_score,curve,BestNet]=SSA(pop,Max_iter,lb,ub,dim,fobj);
```

```
%Draw an evolutionary curve
figure
plot(curve,'r-','linewidth',3)
Xlabel (Evolutionary Algebra)
Ylabel (Root Mean Square Error RMSE)
Legendary ('Best Fit')
Title ('SSA-LSTM Evolutionary Convergence Curve ')

disp('')
Disp (the optimal number of hidden units is', num2str(round(Best_pos(1)))]);
Disp (the optimal maximum training period is', num2str(round(Best_pos(2)))]);
Disp (the optimal initial learning rate is', num2str((Best_pos(3)))]);

%%Modeling each component
for d=1:c
Disp (['di ', num2str(d), 'Component Modeling'

X_imf=[X(:,1:end-1) imf(d,:)'];

%Refactoring the dataset
for i = 1: num_samples - kim - zim + 1
res(i, :) = [reshape(X_imf(i: i + kim - 1,:), 1, kim*or_dim), X_imf(i + kim + zim - 1,:)];
end


%Division of training and testing sets
outdim = 1; %The last column is the output
```

```
num_size = 0.7; %Proportion of training set to dataset
num_train_s = round(num_size * num_samples); %Number of training set samples
f_ = size(res, 2) - outdim; %Input feature dimension



P_train = res(1: num_train_s, 1: f_)';
T_train = res(1: num_train_s, f_ + 1: end)';
M = size(P_train, 2);

P_test = res(num_train_s + 1: end, 1: f_)';
T_test = res(num_train_s + 1: end, f_ + 1: end)';
N = size(P_test, 2);

%Data normalization
[p_train, ps_input] = mapminmax(P_train, 0, 1);
p_test = mapminmax('apply', P_test, ps_input);

[t_train, ps_output] = mapminmax(T_train, 0, 1);
t_test = mapminmax('apply', T_test, ps_output);

%Format conversion
for i = 1 : M
vp_train{i, 1} = p_train(:, i);
vt_train{i, 1} = t_train(:, i);
end

for i = 1 : N
```

```
vp_test{i, 1} = p_test(:, i);

vt_test{i, 1} = t_test(:, i);

end


%LSTM prediction of optimal parameters

layers = [ ...

SequenceInputLayer (f_)% Input Layer

LstmLayer (round (Best_pos (1))% LSTM layer

ReluLayer% Relu activation layer

Fully ConnectedLayer (outdim)% Regression Layer

regressionLayer];

Options=trainingOptions ('adam ',...% optimization algorithm Adam

'MaxEpochs', round(Best_pos(2)),    . ..% maximum training times

'GradientThreshold',    1, . ..% gradient threshold

'InitialLearnRate', Best_pos(3),    . ..% initial learning rate

'LearnRateSchedule', 'piecewise',    . Adjustment of learning rate

'LearnRateDropPeriod', round(Best_pos(2)*0.9),    . Adjust learning rate after 850 training
sessions

'LearnRateDropFactor', 0.2, . ..% learning rate adjustment factor

'L2Regularization',    0.001, . ..% regularization parameter

'ExecutionEnvironment', 'cpu', ...% Training Environment

'Verbose', 0 ..% Close optimization process

'Plots', 'training-progress'); %Draw a curve


%Training

net = trainNetwork(vp_train, vt_train, layers, options);

%Prediction
```

```
t_sim7 = predict(net, vp_train);
t_sim8 = predict(net, vp_test);

%Data de normalization
T_sim7_imf = mapminmax('reverse', t_sim7, ps_output);
T_sim8_imf = mapminmax('reverse', t_sim8, ps_output);

%Data format conversion
T_sim7(d,:) = cell2mat(T_sim7_imf); %Cell2mat converts cell arrays into regular arrays
T_sim8(d,:) = cell2mat(T_sim8_imf);
T_train7(d,:)= T_train;
T_test8(d,:)= T_test;
end

%Add up the predicted results of each component
T_sim7=sum(T_sim7);
T_sim8=sum(T_sim8);
T_train7=sum(T_train7);
T_test8=sum(T_test8);

%Indicator calculation
Disp ('Training Set Error Indicator')
[mae7,rmse7,mape7,error7]=calc_error(T_train7,T_sim7);
fprintf('\n')

Disp ('Test Set Error Indicator')
[mae8,rmse8,mape8,error8]=calc_error(T_test8,T_sim8);
```

```
fprintf('\n')
toc

%%Comparison of Four Model Training Set Results Plots

figure
plot(T_train1,'k','linewidth',3);
hold on;
plot(T_sim1,'m','linewidth',3);
hold on;
plot(T_train5,'g','linewidth',3);
hold on;
plot(T_sim7,'r','linewidth',3);
legend('Target','LSTM','VMD-LSTM','VMD-SSA-LSTM');
%title('Comparative diagram of training set results of three prediction models');
xlabel('Sample Index');
ylabel('Values');
grid on;


figure
plot(T_train1-T_sim1','k','linewidth',3);
hold on
plot(T_train1-T_train5,'g','linewidth',3);
hold on
plot(T_train1-T_sim7,'r','linewidth',3);
legend('LSTM','VMD-LSTM','VMD-SSA-LSTM');
```

```
%title('Comparative diagram of training set error results of three prediction models');
grid on;


%%%%%%%%%%%%%%%%%%%%%%%%%%%%%%%%%%%%%%

%%Comparison of Four Model Test Set Results Plots




figure
plot(T_test2,'k','linewidth',3);
hold on;
plot(T_sim2,'y','linewidth',3);
hold on;
plot(T_sim6,'g','linewidth',3);
hold on;
plot(T_sim8,'r','linewidth',3);
legend('Target','LSTM','VMD-LSTM','VMD-SSA-LSTM');
%title('Comparative diagram of test set results of three prediction models');
xlabel('Sample Index');
ylabel('Values');
grid on;

figure
plot(error2,'k','linewidth',3);
```

```
hold on
plot(error6,'g','linewidth',3);
hold on
plot(error8,'r','linewidth',3);
legend('LSTM','VMD-LSTM','VMD-SSA-LSTM');
%title('Comparative diagram of test set results of three prediction models');
grid on;
```
