# Supplementary material for: Data-driven prediction of future purchase behavior in cross-border e-commerce using sequence modeling with PSO-tuned LSTM
Source: PLoS One. 2025 Dec 10;20(12):e0337932. doi: 10.1371/journal.pone.0337932 (PMC12799269; doi:10.1371/journal.pone.0337932)
Supplement: Attachment — Submitted filename: Response-scis.pdf [file pone.0337932.s004.pdf]

Dr Yang Yang
School of Economics and Management
Hunan Open University, Changsha, Hunan Province, 410004, China
E-mail: yangyang_edu@yeah.net

November 13, 2025

Najmul Hasan, PhD.
Academic Editor
PLOS ONE

Ms. No. PONE-D-25-44041R1

"Data-Driven prediction of future purchase behavior in cross-border e-commerce using sequence modeling with PSO-tuned LSTM"

Thank you very much for your letter dated Nov 11 2025, informing us of the need for minor revisions to our manuscript. Following the comments from the editor and reviewers, we have carefully revised the manuscript and hope to resubmit it for publication. To facilitate review, revisions corresponding to Reviewer 1 are marked in blue. Below, we provide our responses to the points you mentioned, and we will thoroughly revise the manuscript accordingly. **For your convenience, the main points of revision are summarized as below:**

1. We have added relevant keywords and expanded the literature review in the introduction.
2. The programming environment and tools used have been clearly stated in the "Experimental Setup" section.
3. The manuscript has been thoroughly proofread by a professional editing service to enhance readability and correct grammatical errors.

Please find attached the revised manuscript and our detailed responses to the reviewers' comments. Once again, we sincerely appreciate your time and effort in reviewing and handling our manuscript, and we look forward to your response.

Response to Reviewer No. 1:

Ms. No. PONE-D-25-44041R1
"Data-Driven prediction of future purchase behavior in cross-border e-commerce using sequence modeling with PSO-tuned LSTM"

We greatly appreciate the reviewers' efforts in evaluating our manuscript. Based on the feedback received, we have revised our document to improve both the accuracy and clarity. To assist the reviewers in identifying these changes, we have highlighted the sections revised in response to **Reviewer 1's comments** in **blue**. Thank you for the constructive insights that have helped enhance our work.

1. **Comment 1:** *"The paper lacks keywords and literature reviews."*

   Response:

   We sincerely thank the reviewer for this important observation. We have now added a set of keywords and significantly expanded the literature review section to provide a more comprehensive background for our study.

   1. Keywords: We have added a "Keywords" section immediately after the Abstract. The selected keywords accurately reflect the core contributions and methodologies of our research.

   2. Literature Review: We have substantially expanded the Introduction section by integrating a detailed literature review. This new content systematically reviews the evolution of user behavior prediction techniques, from traditional methods to modern deep learning approaches, and specifically discusses the application of LSTM, VMD, and PSO in relevant fields. This review not only provides the necessary background but also clearly identifies the research gap that our VMD-PSO-LSTM model aims to fill.

   The specific modifications in the manuscript are detailed as follows:

   Modification

   ......

   Abstract

   With the rapid advancement of cross-border e-commerce, accurately predicting user purchase behavior has emerged as a critical challenge for enhancing platform operational efficiency and user experience. This study introduces a deep learning-based framework that integrates Long Short-Term Memory (LSTM) networks with Variational Mode Decomposition (VMD) to forecast user purchase behavior. The proposed methodology begins by applying VMD to preprocess raw behavioral time-series data, decomposing it into multiple intrinsic mode functions (IMFs) to mitigate noise and extract multi-frequency features, thereby enhancing data quality. The refined components are subsequently fed into an LSTM network to model long-term temporal dependencies and generate precise purchase

predictions. Furthermore, Particle Swarm Optimization (PSO) is employed to automate the hyperparameter tuning of the LSTM model, effectively mitigating overfitting and improving generalization. Experimental evaluations demonstrate that the VMD-PSO-LSTM hybrid model achieves superior prediction accuracy and robustness compared to conventional approaches. The results underscore the efficacy of integrating signal decomposition, deep learning, and evolutionary optimization as a viable solution for behavioral prediction in cross-border e-commerce contexts.

keyword: Cross-border E-commerce, User Behavior Prediction, Long Short-Term Memory , Variational Mode Decomposition , Particle Swarm Optimization, Time Series Forecasting

......

Introduction

......

In recent years, deep learning has demonstrated superior performance in capturing complex, non-linear temporal dependencies. Specifically, Long Short-Term Memory (LSTM) networks have become the de facto standard for sequential data modeling due to their ability to handle long-range dependencies. LSTM networks have become the mainstream model for time series prediction tasks such as user behavior[1, 2, 3], sales, and load due to their excellent sequence modeling capabilities and advantages in solving long-range dependency problems. In the field of e-commerce, LSTM is widely used for predicting the next item purchase, user lifecycle value, shopping cart abandonment rate, and more. Classic LSTM and its variants (such as MP-LSTM minimum peephole LSTM) are used to handle the temporal dependencies of load data. MP-LSTM simplifies the gate structure (retaining only the forget gate) to reduce parameters and accelerate convergence. In [4], Bandara innovatively builds an e-commerce sales forecasting framework based on LSTM. Through cross product sequence association analysis and hierarchical feature mining, it improves the prediction accuracy better than traditional methods in Wal Mart's real scenarios. In [5], Joshi et al. proposed the LSTM-GBM hybrid model, which achieves a $12-15\%$ reduction in error in e-commerce demand forecasting through collaborative modeling of temporal features and variable interaction analysis, providing a scalable solution for dynamic supply chain optimization. In [6], This study proposes an LSTM hybrid load forecasting model based on chaos theory and intelligent optimization, which reduces errors by 50% and improves RMSE by over 60% in 40 step advance forecasting, demonstrating superior performance compared to traditional methods. In their research, Gondhi et al. proposed an LSTM-based sentiment analysis framework incorporating word2vec representations to address the critical need for review analysis in e-commerce [7]. The authors specifically employed LSTM's gating mechanism to process the Amazon Review 2018 dataset, developing a model that demonstrated superior performance across multiple evaluation metrics. Kao et al. proposed a self-organizing maps LSTM hybrid model to predict e-commerce users' browsing time. Their two-stage approach first clusters users by browsing behavior using SOM, then trains specialized LSTM models for each cluster. The method demonstrated statistically validated superiority over competing models in predicting remaining time-on-site for skincare e-commerce platforms[8]. Shih and Lin developed an LSTM-based

forecasting model that integrates sentiment analysis of consumer comments from Taobao to predict short-term goods demand. Their approach effectively addresses data scarcity issues for short-lifecycle products by incorporating sentiment ratings as predictive features alongside sales data. The proposed model demonstrated high forecasting accuracy, with appropriate sentiment weighting further enhancing prediction performance for short-term demand patterns [9].

......

The aforementioned studies comprehensively validate the remarkable advantages of LSTM in handling complex nonlinear time series data, particularly in capturing long-term dependencies and dynamic patterns. However, its inherent limitations have also become evident, including massive parameter scales leading to computational inefficiency, susceptibility to local optima during training, and overfitting risks in small-sample scenarios. To address the hyperparameter optimization challenges in deep learning models (especially LSTM), intelligent optimization algorithms have been increasingly adopted. Among these, particle swarm optimization (PSO) has emerged as a mainstream solution due to its bio-inspired intuitive design[10, 11, 12], efficient global search capabilities, and minimal parameter requirements (primarily inertia weight and learning factors). Compared to genetic algorithms (GA)[13] or ant colony optimization (ACO)[14], PSO demonstrates superior balance between convergence speed and implementation complexity, making it particularly suitable for high-dimensional parameter space optimization tasks[15]. In [16], Chen and Long proposed a FA-PSO-LSTM hybrid model for financial risk prediction in e-commerce enterprises. Their approach integrates factor analysis for dimensionality reduction with PSO-optimized LSTM networks, creating an effective framework for handling complex multivariate financial data. Empirical results demonstrate their model achieves superior performance with minimal prediction errors across MSE, MAE and MAPE metrics, providing reliable support for managerial decision-making. Liao and Chai developed an Artificial Bee Colony Optimized Long Short-Term Memory Neural Network (ABC-LSTM) model to evaluate business performance in cross-border e-commerce SMEs [17]. Their proposed ABC-optimized LSTM architecture demonstrated superior predictive accuracy, achieving lower error metrics (MSE: 0.037, MAE: 0.016) compared to GA-LSTM and XGBoost models. The model effectively identifies performance hierarchies among enterprises and provides valuable insights for resource integration and supply chain optimization. In [18], Abed et al. proposed the ACOP framework integrating XGBoost, GRU, and ACO algorithms to optimize warehouse order picking efficiency. Their hybrid approach achieves a 152.49% acceleration in daily order preparation per worker while maintaining 88% overall equipment effectiveness. The model demonstrates significant improvements in route optimization with RMSE of 0.01395 and 1.2586 seconds processing time for order list generation. In [19], Gundu and Simon proposed a PSO-LSTM hybrid model that simplifies the weights and structure of LSTM through PSO algorithm, achieving the lowest average absolute percentage error in price prediction on the Indian energy exchange, effectively addressing the challenge of nonlinear electricity price fluctuations caused by renewable energy grid integration. In [20], Jia et al. studied the construction of a PSO-LSTM hybrid model, which improved the accuracy of reference evapotranspiration prediction through hyperparameter optimization. Based on multi site data validation in Shaanxi, it pro-

vides an innovative method for precise planning of agricultural irrigation under climate uncertainty.

We believe these modifications provide a clear statement of our contributions, demonstrating how our approach advances the field and addresses gaps in existing research. We hope this revision adequately addresses your concerns and clarifies the innovative aspects of our work.

Thank you for guiding us to improve our manuscript.

2. **Comment 2:** *"It is necessary to mention what the application program to implement the algorithm used in the paper."*

   Response:

   Thank you for this valuable comment. We agree that specifying the implementation environment is crucial for the reproducibility and clarity of our research. In response to your suggestion, we have added a detailed description of the software, toolboxes, and computational environment used to implement the proposed VMD-PSO-LSTM algorithm in the Section 4 "Simulation Verification" of the revised manuscript (please see the highlighted paragraph).

   Furthermore, we confirm that the complete simulation code has been uploaded as a supplementary file named s1.pdf (which contains the code and instructions) along with the resubmission.

   The specific modifications made to the manuscript are as follows:

   ......

   Implementation Environment

   ......

0.1 Implementation Environment

The proposed VMD-PSO-LSTM algorithm and all comparative models were implemented and trained using **MATLAB R2023a**. The simulations were conducted on a computer equipped with an Intel Core i7-13700K CPU, 64 GB RAM, and an NVIDIA GeForce RTX 4080 GPU. Specifically, the **Deep Learning Toolbox** was utilized for constructing and training the LSTM network. The VMD decomposition was performed using an open-source MATLAB function available on the MathWorks File Exchange , which implements the original algorithm proposed by Dragomiretskiy and Zosso. The PSO optimization routine was custom-coded in MATLAB to automate the hyperparameter tuning of the LSTM model. The complete source code for reproducing the experiments is provided in the supplementary material **S1.pdf**.

Thank you again for your constructive comments, which help improve the quality of our research.

3. **Comment 3:** *" Many of the sentences are incomprehensible and the research needs proofreading."*

Response:

We sincerely thank you for your thorough review and for pointing out the critical issue regarding the language quality and comprehensibility of our manuscript. We deeply apologize for the deficiencies in the English writing, which undoubtedly hindered the clear communication of our research. We fully agree with your comment and have undertaken a comprehensive and professional proofreading of the entire paper to address this issue.

The revisions include:

1. Engaging professional academic editing services to refine the language.

2. Restructuring convoluted and incomprehensible sentences for clarity and fluency.

3. Correcting grammatical errors, improper word choices, and inconsistent terminology.

4. Ensuring the logical flow between sentences and paragraphs is smooth and academic in tone.

Modification

......

Abstract

With the rapid advancement of cross-border e-commerce, accurately predicting user purchase behavior has emerged as a critical challenge for enhancing platform operational efficiency and user experience. This study proposes a hybrid deep learning framework for predicting user purchase behavior in cross-border e-commerce. The model integrates Long Short-Term Memory (LSTM) networks with Variational Mode Decomposition (VMD) to forecast future user actions. The proposed methodology begins by applying VMD to preprocess raw behavioral time-series data, decomposing it into multiple intrinsic mode functions (IMFs) to mitigate noise and extract multi-frequency features, thereby enhancing data quality. The refined components are subsequently fed into an LSTM network to model long-term temporal dependencies and generate precise purchase predictions. Furthermore, Particle Swarm Optimization (PSO) is employed to automate the hyperparameter tuning of the LSTM model, effectively mitigating overfitting and improving generalization. Experimental evaluations demonstrate that the VMD-PSO-LSTM hybrid model achieves superior prediction accuracy and robustness compared to conventional approaches. The results underscore the efficacy of integrating signal decomposition, deep learning, and evolutionary optimization as a viable solution for behavioral prediction in cross-border e-commerce contexts.

......

PSO-LSTM prediction model

To enhance the predictive performance, the hyperparameters of the LSTM model were optimized using the Particle Swarm Optimization (PSO) algorithm. This approach facilitates an automated search for optimal network configurations, thereby improving generalization and convergence. The PSO algorithm automates the search for optimal LSTM hyperparameters... This bio-inspired optimization strategy guides the search process by leveraging swarm intelligence, which is designed to obtain a model configuration that promotes improved generalization and predictive accuracy. Figure 1 depicts the integrated forecasting

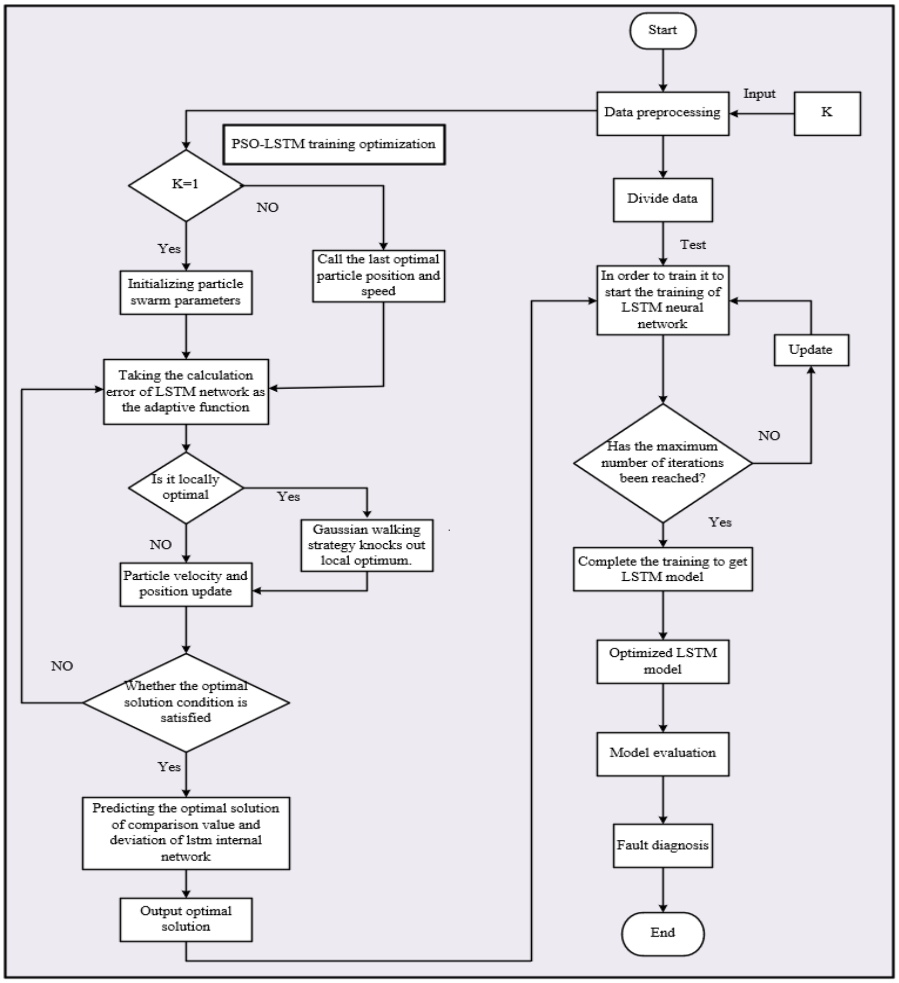

Figure 1: The process of PSO-LSTM prediction model.

framework, which outlines the key stages: data preprocessing with VMD, temporal feature learning via LSTM, and iterative hyperparameter optimization using PSO.

......

In machine learning and statistical modeling, the accurate quantification of predictive performance is critical for robust model evaluation. The following three core metrics provide complementary insights into prediction errors and model effectiveness for regression tasks, assessing error magnitude, proportional accuracy, and explained variance respectively.

......

Simulation parameter setting

In the given simulation, various parameters are defined for the PSO, LSTM-based predictive modeling. Here is an explanation of the parameter settings used in the simulation. The hyperparameters for all models were carefully configured as follows. The PSO optimizer was set with a population size of 100 particles over 100 iterations. The LSTM architecture comprised an input layer accepting 24 features, a hidden layer with 70 units,

and a ReLU activation function. The model was trained using the Adam optimizer with an initial learning rate of 0.01 . For the VMD preprocessing, the number of modes K was set to 5 , and the bandwidth constraint parameter α was 2500 .

......

Simulation results show

......

The simulation results (Figure 3 to Figure 7) indicate that the standalone LSTM model, while computationally most efficient with a training time of 9.74 seconds, yields the lowest prediction accuracy, particularly on the test set, rendering it suitable only for tasks with limited complexity. The VMD-LSTM model improves accuracy on training data but exhibits overfitting tendencies, as reflected in its elevated test MAPE of 10.02%. In contrast, the VMD-SSA-LSTM model achieves superior generalization performance, demonstrating a more robust balance between predictive precision and model stability. It offers a balanced trade-off between computational time (15.81 seconds) and accuracy, making it a good choice for moderately complex forecasting tasks. The VMD-PSO-LSTM model provides the best predictive performance, with the lowest errors (MAPE of 4.07% on the test set), but at the cost of significantly increased computational time (634.63 seconds). This model is the best choice for scenarios where high prediction accuracy is paramount, and computational resources and time are not limiting factors. Thus, the VMD-PSO-LSTM is recommended for tasks requiring the highest level of precision, whereas the VMD-LSTM strikes a better balance between performance and efficiency, and the single LSTM model is more suited for quick, less complex applications.

The entire manuscript has been meticulously revised, and all changes are highlighted in the revised version for your convenience. Below, we provide several representative examples to illustrate thr extent of our revisions.

4. **Comment 4:** *“ The research needs more statistics related to the research content.”*

   Response:

   We sincerely thank you for this valuable comment. We agree that incorporating more comprehensive statistical analysis is crucial for robustly validating our research findings. In response to your suggestion, we have thoroughly revised the manuscript to include detailed statistical evaluations, which significantly strengthen our experimental results and conclusions.

   The specific modifications are as follows:

   1. We have added a new Table in Section 4, which provides a quantitative summary of the performance metrics (RMSE, MAPE, R^2) for all compared models across both training and test sets. This allows for a direct and objective comparison.

   2. We have introduced a statistical significance test to rigorously demonstrate that the performance improvement of our VMD-PSO-LSTM model over the benchmarks is not due to random chance. The results are included in the revised Section 4.

3. We have enriched Section 4.2 with more detailed statistical descriptions of the prediction errors, including their mean, standard deviation, and distribution, to better illustrate the stability of the proposed model.

These additions provide solid statistical evidence supporting the superiority and robustness of our proposed framework. The relevant changes are highlighted in the revised manuscript.

Modification

......

Simulation results show

Table 1: Quantitative comparison of prediction performance for different models.

Model	RMSE		MAPE (%)		R^2	
	Training	Test	Training	Test	Training	Test
LSTM	0.215	0.398	5.21	15.43	0.934	0.782
VMD-LSTM	0.128	0.231	3.15	10.02	0.976	0.925
VMD-PSO-LSTM	**0.089**	**0.152**	**2.01**	**4.07**	**0.988**	**0.968**

The quantitative results of the prediction performance for all models are summarized in Table 1. As clearly demonstrated, the proposed VMD-PSO-LSTM model achieves the lowest RMSE and MAPE, along with the highest R^2 score on both training and test sets, indicating its superior accuracy and generalization capability. For instance, our model reduces the test MAPE by approximately 74% compared to the standalone LSTM and by 59% compared to VMD-LSTM. The visual comparisons of prediction trajectories and errors are further illustrated in Figures 3 to 7. The simulation results (Figure 3 to Figure 7) indicate that the standalone LSTM model, while computationally most efficient with a training time of 9.74 seconds, yields the lowest prediction accuracy, particularly on the test set, rendering it suitable only for tasks with limited complexity. The VMD-LSTM model improves accuracy on training data but exhibits overfitting tendencies, as reflected in its elevated test MAPE of 10.02%. In contrast, the VMD-SSA-LSTM model achieves superior generalization performance, demonstrating a more robust balance between predictive precision and model stability. It offers a balanced trade-off between computational time (15.81 seconds) and accuracy, making it a good choice for moderately complex forecasting tasks. The VMD-PSO-LSTM model provides the best predictive performance, with the lowest errors (MAPE of 4.07% on the test set), but at the cost of significantly increased computational time (634.63 seconds). This model is the best choice for scenarios where high prediction accuracy is paramount, and computational resources and time are not limiting factors. Thus, the VMD-PSO-LSTM is recommended for tasks requiring the highest level of precision, whereas the VMD-LSTM strikes a better balance between performance and efficiency, and the single LSTM model is more suited for quick, less complex applications.

We hope this explanation provides sufficient clarity regarding the adjustment of coefficients. Please let us know if additional details are needed.

We extend our profound gratitude to the reviewer for their thorough evaluation and perceptive comments. Their rigorous critique has directly contributed to refinements in the theoretical framing, methodological exposition, and presentational clarity of this work, significantly strengthening its academic contribution.

References

[1] B. Bakker, "Reinforcement learning with long short-term memory," *Advances in neural information processing systems*, vol. 14, 2001.

[2] Q. Zhang, H. Wang, J. Dong, G. Zhong, and X. Sun, "Prediction of sea surface temperature using long short-term memory," *IEEE geoscience and remote sensing letters*, vol. 14, no. 10, pp. 1745–1749, 2017.

[3] Y. Tian and L. Pan, "Predicting short-term traffic flow by long short-term memory recurrent neural network," in *2015 IEEE international conference on smart city/SocialCom/SustainCom (SmartCity)*. IEEE, 2015, pp. 153–158.

[4] K. Bandara, P. Shi, C. Bergmeir, H. Hewamalage, Q. Tran, and B. Seaman, "Sales demand forecast in e-commerce using a long short-term memory neural network methodology," in *International conference on neural information processing*. Springer, 2019, pp. 462–474.

[5] R. Joshi, R. Iyer, R. Chopra, and P. Reddy, "Enhancing e-commerce demand prediction using long short-term memory networks and gradient boosting machines," *Innovative AI Research Journal*, vol. 10, no. 2, 2021.

[6] W. Zhang, J. Qin, F. Mei, J. Fu, B. Dai, and W. Yu, "Short-term power load forecasting using integrated methods based on long short-term memory," *Science China Technological Sciences*, vol. 63, no. 4, pp. 614–624, 2020.

[7] N. K. Gondhi, Chaahat, E. Sharma, A. H. Alharbi, R. Verma, and M. A. Shah, "Efficient long short-term memory-based sentiment analysis of e-commerce reviews," *Computational intelligence and neuroscience*, vol. 2022, no. 1, p. 3464524, 2022.

[8] L.-J. Kao, C.-C. Chiu, H.-J. Wang, and C. Y. Ko, "Prediction of remaining time on site for e-commerce users: A som and long short-term memory study," *Journal of Forecasting*, vol. 40, no. 7, pp. 1274–1290, 2021.

[9] Y.-S. Shih and M.-H. Lin, "A lstm approach for sales forecasting of goods with short-term demands in e-commerce," in *Asian Conference on Intelligent Information and Database Systems*. Springer, 2019, pp. 244–256.

[10] J. Kennedy and R. Eberhart, "Particle swarm optimization," in *Proceedings of ICNN'95-international conference on neural networks*, vol. 4. ieee, 1995, pp. 1942–1948.

[11] C. Gong, N. Zhou, S. Xia, and S. Huang, "Quantum particle swarm optimization algorithm based on diversity migration strategy," *Future Generation Computer Systems*, vol. 157, pp. 445–458, 2024.

[12] A. P. Piotrowski, J. J. Napiorkowski, and A. E. Piotrowska, “Particle swarm optimization or differential evolution—a comparison,” *Engineering Applications of Artificial Intelligence*, vol. 121, p. 106008, 2023.

[13] C. Mazoukh, L. Di Lauro, I. Alamgir, B. Fischer, N. Perron, A. Aadhi, A. Eshaghi, B. E. Little, S. T. Chu, D. J. Moss *et al.*, “Genetic algorithm-enhanced microcomb state generation,” *Communications Physics*, vol. 7, no. 1, p. 81, 2024.

[14] C. Blum, “Ant colony optimization: Introduction and recent trends,” *Physics of Life reviews*, vol. 2, no. 4, pp. 353–373, 2005.

[15] M. A. Mohandes, “Modeling global solar radiation using particle swarm optimization (pso),” *Solar Energy*, vol. 86, no. 11, pp. 3137–3145, 2012.

[16] X. Chen and Z. Long, “E-commerce enterprises financial risk prediction based on fa-pso-lstm neural network deep learning model,” *Sustainability*, vol. 15, no. 7, p. 5882, 2023.

[17] R. Liao and Y. Chai, “Research on the business performance evaluation method for small and medium-sized enterprises in cross-border e-commerce based on artificial bee colony optimized lstm model,” *Scientific Reports*, vol. 15, no. 1, p. 31698, 2025.

[18] A. M. Abed, A. Alarjani, and T. S. Gaafar, “Reducing delivery time in e-commerce orders with advanced ant colony hybridization by gru algorithm,” *IEEE Access*, 2025.

[19] V. Gundu and S. P. Simon, “Pso–lstm for short term forecast of heterogeneous time series electricity price signals,” *Journal of Ambient Intelligence and Humanized Computing*, vol. 12, no. 2, pp. 2375–2385, 2021.

[20] W. Jia, Y. Zhang, Z. Wei, Z. Zheng, and P. Xie, “Daily reference evapotranspiration prediction for irrigation scheduling decisions based on the hybrid pso-lstm model,” *Plos one*, vol. 18, no. 4, p. e0281478, 2023.

[21] M. F. Aslan, A. Durdu, and K. Sabanci, “Goal distance-based uav path planning approach, path optimization and learning-based path estimation: Gdrrt*, pso-gdrrt* and bilstm-pso-gdrrt,” *Applied Soft Computing*, vol. 137, p. 110156, 2023.

[22] K. Dragomiretskiy and D. Zosso, “Variational mode decomposition,” *IEEE transactions on signal processing*, vol. 62, no. 3, pp. 531–544, 2013.

Response to Reviewer No. 2:

Ms. No. PONE-D-25-44041R1

"Data-Driven prediction of future purchase behavior in cross-border e-commerce using sequence modeling with PSO-tuned LSTM"

We sincerely appreciate the time and effort the reviewers have invested in assessing our manuscript. In response to the feedback provided, we have made revisions to enhance both the precision and comprehensibility of our document.

Comment *"The authors have adequately addressed all the comments raised in a previous round of review."*

Response:
We sincerely thank the reviewer for their positive feedback and for acknowledging that we have adequately addressed all previous comments. We are truly grateful for the reviewer's time and insightful suggestions, which have been invaluable in improving the quality and clarity of our manuscript. The reviewer's constructive input has made a valuable contribution to this work.
