# Supplementary material for: Data-driven prediction of future purchase behavior in cross-border e-commerce using sequence modeling with PSO-tuned LSTM
Source: PLoS One. 2025 Dec 10;20(12):e0337932. doi: 10.1371/journal.pone.0337932 (PMC12799269; doi:10.1371/journal.pone.0337932)
Supplement: Attachment — Submitted filename: Data.docx [file pone.0337932.s003.docx]

Data-Driven prediction of future purchase behavior in
cross-border e-commerce using sequence modeling with
PSO-tuned LSTM

1. The paper lacks keywords and literature reviews.
2. It is necessary to mention what the application program to implement the algorithm used in the paper.
3. Many of the sentences are incomprehensible and the research needs proofreading.
4. The research needs more statistics related to the research content
